# Global and China lower respiratory infection burden in children under 5 years: implications for vaccine equity and pediatric clinical care

**DOI:** 10.3389/fmed.2026.1971991

**Published:** 2026-10-22

**Authors:** Xingyue Su, Die Liu, Jing Liu, Di Lv, Jianning Guo, Qin Hui, Fang Ye, Yuanmei Chen, Chao Wang, Lijuan Tang, Meihong Xia, Qi Zhang

**Affiliations:** 1 Department of Pediatrics, China-Japan Friendship Hospital (Institute of Clinical Medical Sciences), Beijing, China; 2 Chinese Academy of Medical Sciences and Peking Union Medical College, Beijing, China

**Keywords:** child health, global burden of disease, lower respiratory infection, pediatric pneumonia, pneumococcal disease, respiratory support, vaccine equity

## Abstract

**Background:**

Lower respiratory infection (LRI) remains an important cause of death and health loss in children younger than 5 years. We examined the residual global and Chinese under-5 LRI burden, vaccine-relevant pathogen inequalities, and the separate clinical-care needs of children hospitalized with pneumonia in China.

**Methods:**

GBD and related public data were used to characterize under-5 LRI incidence, deaths, DALYs, SDI gradients, pathogen-attributed burden, and national Hib3 and PCV3 coverage. A separate single-center pediatric pneumonia hospitalization cohort from China-Japan Friendship Hospital (July 1, 2024 to December 31, 2025) characterized clinical severity, respiratory support, length of stay, and pathogen testing among children aged 0 to 4 years. The components were analyzed in parallel, were not pooled, and were not used to validate one another.

**Results:**

From 1990 to 2021, global under-5 LRI deaths declined from 1,898,507 to 503,409, and DALYs declined from 169,370,501 to 44,882,094. GBD 2023 estimates for China showed 2,895,598 incident under-5 LRI cases, 4,859 deaths, and 436,398 DALYs. In 2021, Streptococcus pneumoniae caused 166,379 under-5 LRI deaths, representing 33.1% of all such deaths. Streptococcus pneumoniae and the Haemophilus influenzae proxy together accounted for 81.0% of the pathogen-specific mortality-rate gap between the lowest and highest SDI quintiles. The hospital dataset included 1,717 pediatric pneumonia records, including 889 children aged 0 to 4 years. In this subgroup, 170 (19.1%) were recorded as severe, 184 (20.7%) received respiratory support, and 92 (10.3%) received high-flow oxygen therapy, non-invasive ventilation, or invasive mechanical ventilation.

**Conclusions:**

Global under-5 LRI burden has declined substantially, but residual burden remains concentrated in lower-SDI settings and is weighted toward vaccine-relevant bacterial pathogens. Separate hospitalization data from China show persistent severe-disease and respiratory-support needs among young children. Childhood LRI control should address vaccine equity alongside early recognition, oxygen capacity, and inpatient pneumonia management.

## Introduction

Lower respiratory infection remains an important infectious cause of childhood death and health loss, with children younger than 5 years being especially affected. Recent GBD work has shown that global LRI mortality and DALY rates have continued to decline since 1990, but LRI still occupies an important place among infectious causes of death worldwide and continues to cause health loss in children (1). Independent systematic estimates of childhood pneumonia have also shown declines in incidence and mortality among children younger than 5 years from 2000 to 2015, yet the burden remains substantial and prevention, early recognition, and standardized treatment still need sustained strengthening (2). The key question now is therefore not only whether the burden has fallen, but where the burden remaining after that decline is concentrated, which pathogens are involved, and which parts of the health system remain weak (3–5).

Although the overall burden of childhood LRI has continued to decline, risk reduction has not occurred synchronously across countries and regions. Pneumococcal conjugate vaccines (PCVs) and *Haemophilus influenzae* type b (Hib) vaccines have shown effects in reducing invasive disease and pneumonia-related burden across several study designs (6–13). However, countries still differ substantially in timing of vaccine introduction, completion of full immunization schedules, and sustained coverage. Low-resource settings often face both higher infection risk and more limited health-service capacity (14–17). If the remaining under-5 LRI burden is still concentrated mainly in vaccine-relevant bacterial pathogens and low-coverage settings, this pattern is not only a matter of pathogen distribution. It also indicates that immunization equity and pediatric pneumonia care capacity remain central to further burden reduction.

GBD and related public data are more suitable for understanding the population-level burden of LRI. Through relatively standardized disease definitions and modelling frameworks, GBD provides long-term, cross-nationally comparable data on incidence, deaths, DALYs, Socio-demographic Index (SDI) gradients, and pathogen-attributed burden (1, 18). WUENIC and WHO vaccine-introduction data provide country-level information on Hib3 and PCV3 coverage and vaccine introduction timing (19, 20). These data allow assessment of whether LRI burden, pathogen attribution, and immunization coverage gaps show corresponding distributions at the country level. However, GBD is a set of modelled population-level estimates and cannot reflect the specific clinical state of hospitalized children at admission, such as disease severity, oxygen or ventilatory support, length of stay, inflammatory biomarkers, and hospital pathogen test results.

Hospitalized pneumonia data can add clinical information that population estimates cannot show. The PERCH study found that the etiologic composition of severe childhood pneumonia requiring hospital admission in Africa and Asia was heterogeneous, with RSV making an important contribution (21). The EPIC study in the United States also suggested that hospitalized community-acquired pneumonia in children was more concentrated among younger children, and that viruses were detected more often than bacteria in radiographically confirmed cases (22). Studies of pediatric community-acquired pneumonia in China have shown that *M. pneumoniae*, RSV, influenza virus, adenovirus, *S. pneumoniae*, and other respiratory viruses may all contribute to childhood pneumonia, with roles varying by age, region, and season (23–26). Hospital pathogen test results should therefore be understood as laboratory findings in particular hospitalized and tested populations; their meaning is shaped by the tested population, test panels, timing of testing, and documentation practice. They cannot be directly extrapolated as population infection rates or national pathogen-attributed burden. Nevertheless, hospitalization data can show the severity, care needs, and commonly detected pathogens after childhood LRI has entered the clinical-care setting.

We addressed a single clinical-public-health question at two levels: where residual under-5 LRI burden and vaccine-relevant inequalities are concentrated, and what pneumonia requires once children reach hospital care. The GBD analyses describe global and Chinese under-5 LRI burden, SDI-related inequalities, pathogen-attributed burden, and immunization coverage gaps. The independent single-center Chinese hospitalization cohort describes disease severity, respiratory-support needs, length of stay, and pathogen testing among children hospitalized with pneumonia. The data sources have different origins, denominators, and scopes of inference; they were not pooled into one statistical sample and were not used to validate each other. Their parallel interpretation identifies complementary priorities for childhood LRI control: vaccine access and coverage equity, early recognition, oxygen support, and inpatient management.

## Materials & methods

This study used two parallel data components. GBD and related public data were used to assess population burden, pathogen attribution, SDI-related differences, and immunization coverage for LRI among children younger than 5 years. An independent single-center pediatric pneumonia hospitalization cohort from China was used to describe age composition, disease severity, respiratory support, length of stay, and pathogen testing among hospitalized children. The components were not pooled and were not designed as a validation study. For the GBD component, we followed GATHER principles. For the hospitalization-record component, we followed STROBE principles (27, 28).

The population-burden analysis was based on GBD country-year under-5 LRI indicators. The global trend analysis covered 1990 to 2021. The China analysis used GBD 2023 China results within the same indicator framework to reflect a more recent national burden level. In addition to GBD, we used WHO Global Health Estimates, UN-IGME child mortality estimates, WUENIC, WHO vaccine-introduction data, UNICEF indicators, and World Bank indicators to provide contextual information on mortality and child survival, immunization coverage, vaccine-introduction timing, and national development (19, 20, 29, 30).

The comparison between GBD and WHO Global Health Estimates was intended to assess whether different public mortality data sources were directionally consistent in overall trends and regional differences, rather than to treat either source as an absolute standard. SDI quintile groups were fixed according to 2021 GBD SDI values and kept constant across the 1990 to 2021 analysis period, reducing the effect of time-varying country grouping on long-term inequality comparisons.

The Results section first presents population-level burden and inequality patterns, then separately describes clinical severity, respiratory-support needs, and pathogen testing among young children hospitalized with pneumonia in China.

### Population outcomes, pathogens, and immunization indicators

The main population-burden indicators were under-5 LRI incidence, deaths, DALYs, age-standardized mortality rate, and age-standardized DALY rate. Pathogen analyses focused on *S. pneumoniae*, *H. influenzae*-related burden, RSV, and influenza virus. *S. pneumoniae* and *H. influenzae* were used to reflect vaccine-relevant bacterial LRI burden, whereas RSV and influenza virus were used to reflect major viral LRI burden.

Immunization analyses used national-year Hib3 and PCV3 coverage. Hib3 and PCV3 coverage were treated as population-level indicators of immunization level and were used to examine ecological associations between coverage and the corresponding pathogen-related burden. Vaccine-introduction timing and coverage differences were summarized by SDI quintile.

### Hospital pneumonia data

The independent clinical component used pediatric pneumonia hospitalization records from China-Japan Friendship Hospital. We included pediatric pneumonia hospitalizations identified between July 1, 2024 and December 31, 2025, and extracted age, sex, disease severity, respiratory support, length of stay, selected treatment orders, laboratory biomarkers, and pathogen-related records. Because GBD mainly focuses on children younger than 5 years, children aged 0 to 4 years in the hospital cohort were prespecified as the main clinical subgroup; older children described the overall hospital case mix of pediatric pneumonia.

Respiratory support was summarized in two categories: receipt of at least one form of respiratory support, and receipt of advanced respiratory support. Advanced respiratory support was defined as high-flow oxygen therapy, non-invasive ventilation, or invasive mechanical ventilation. Pathogen positivity was defined as the number of hospitalization records positive for the relevant test divided by the number of hospitalization records that completed that test.

### Ethics approval and data protection

The hospital clinical-record component was conducted under approval from the Clinical Research Ethics Committee of China-Japan Friendship Hospital (approval No. 2025-KY-033), within the multicenter project approved by the Medical Ethics Committee of Beijing Children's Hospital, Capital Medical University (approval No. 2024-Y-321-D). Written informed consent was obtained from parents or legal guardians, with assent from children aged 8 years or older where applicable, according to the approved consent documents. Names, medical record numbers, and other direct patient identifiers were removed before analysis. Public population-data analyses used de-identified information from established data providers.

### Statistical analysis

Population-burden analyses were primarily descriptive. We summarized under-5 LRI incidence, deaths, DALYs, and corresponding rates by year, country, and SDI group, and presented both global long-term trends and the recent Chinese context. SDI-related inequalities were summarized mainly using the gaps in age-standardized mortality and DALY rates between the lowest and highest SDI quintiles, with additional population-health inequality metrics including the slope index of inequality and the concentration index (30–32).

Pathogen-specific analyses compared deaths and DALYs attributable to *S. pneumoniae*, the *H. influenzae* proxy, RSV, and influenza virus across years and SDI groups. Ecological associations between vaccine coverage and pathogen-related burden were assessed using country and year fixed-effects models with 0-, 1-, 2-, and 3-year lags. Standard errors were clustered by country. The prespecified main lag was 1 year. For the 16 tests formed by two vaccines, two outcome classes, and four lags, we reported nominal *P* values, Benjamini-Hochberg false-discovery-rate correction, and Bonferroni correction. Because vaccine introduction often occurs alongside health-system improvement, socioeconomic change, and long-term trends, fixed-effects and vaccine-introduction analyses were interpreted as population-level associations and sensitivity checks rather than causal effect estimates (31–34).

Burden trends and inequality analyses included GBD country-year data for 204 countries and territories from 1990 to 2021. Fixed-effects models required availability of the relevant vaccine coverage, pathogen-related outcome, and country-year information, and therefore used smaller samples than the full descriptive panel. PCV3 models included 62 countries and 465 country-year observations; Hib3 models included 81 countries and 1,111 country-year observations. Countries lacking reported immunization-program data or sufficient continuous coverage data were retained in descriptive analyses but did not enter the fixed-effects models.

For hospitalization records, categorical variables were summarized as counts and percentages, and continuous variables as medians and interquartile ranges according to distribution; length of stay was presented as a median. Pathogen positivity was calculated among under-5 hospitalization records receiving the relevant test, so denominators could differ across pathogens. Exploratory clinical models used the severe label among 0- to 4-year hospitalization records as the dependent variable to identify severity-related signals in hospitalized cases; results were expressed as odds ratios (ORs) with 95% confidence intervals (CIs). All statistical analyses were performed in R (version 4.5.2); data cleaning and plotting mainly used tidyverse, data.table, and ggplot2. Two-sided *P* values less than 0.05 were considered statistically significant.

## Results

### Global and China under-5 LRI burden trends

Between 1990 and 2021, the global burden of LRI among children younger than 5 years declined markedly. Deaths decreased from 1,898,507 to 503,409, and DALYs decreased from 169,370,501 to 44,882,094. Mean age-standardized mortality and DALY rates also decreased over the same period (Figure 1; Table 1). Extended-year results for 2022 to 2023 and comparison of GBD with WHO Global Health Estimates are provided in Supplementary Figure S1. The 2020 and 2021 time series were affected by changes in health-care seeking, transmission, and estimation frameworks during the COVID-19 pandemic; the long-term trend is therefore interpreted as an overall decline since 1990.

**Figure 1:**
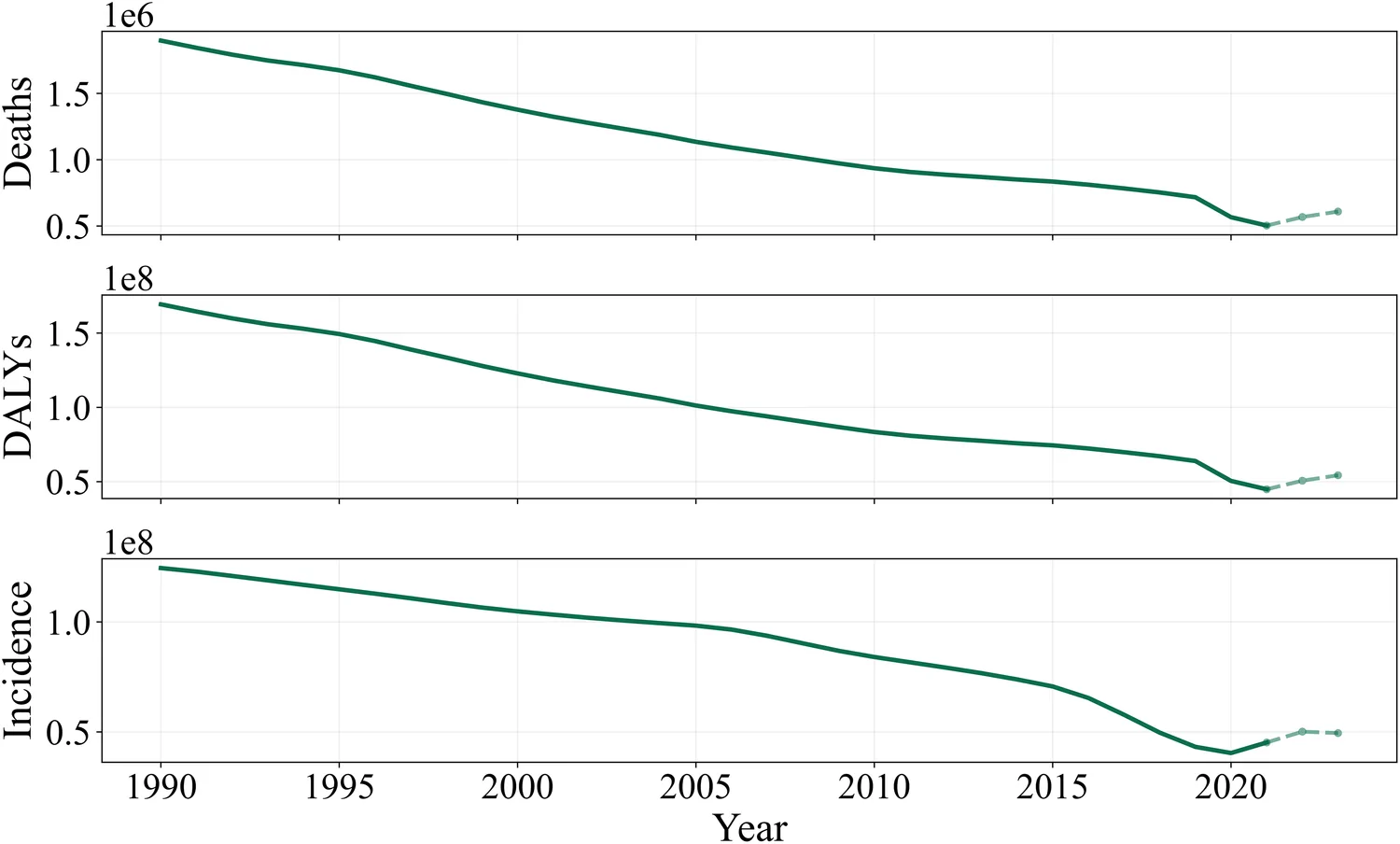
Long-term global under-5 LRI burden trends. Trends in global under-5 LRI deaths, DALYs, and incidence from 1990 to 2021, with dashed extensions for 2022 to 2023. ASDR, age-standardized DALY rate; ASMR, age-standardized mortality rate; DALY, disability-adjusted life-year.

**Table 1:** Global under-5 LRI burden and annualized change.

Year	Countries/territories	Deaths	DALYs	Incidence	Mean ASMR	Mean ASDR	Mean ASIR
1990	204	1,898,507	169,370,501	124,502,882	230.8	20,563.8	14,710.1
2000	204	1,377,432	122,873,473	104,807,795	161.3	14,379.4	12,162.5
2010	204	935,745	83,450,143	84,061,709	99.2	8,844.9	9,240.6
2020	204	566,730	50,520,918	40,418,104	59.0	5,255.7	4,750.9
2021	204	503,409	44,882,094	45,209,165	52.9	4,720.8	5,288.6
1990-2021 EAPC (%)	204				−4.3	−4.3	−3.3

ASDR, age-standardized DALY rate; ASIR, age-standardized incidence rate; ASMR, age-standardized mortality rate; DALY, disability-adjusted life-year; EAPC, estimated annual percentage change.

Chinese data likewise showed that under-5 LRI continued to cause measurable disease burden. GBD 2023 estimates for China showed 2,895,598 incident LRI cases among children younger than 5 years, an incidence rate of 4,658.0 per 100,000, 4,859 deaths, and 436,398 DALYs. These results provide recent national context for the pediatric pneumonia hospitalization data in China, but they were not used to directly extrapolate incidence or mortality from the single-center hospitalization cases.

GBD 2023 pathogen-attribution results for China showed that S. pneumoniae remained the largest source of deaths and DALYs among the pathogens considered in this study, with an estimated 2,162 deaths and 193,740 DALYs. The corresponding death counts for the H. influenzae proxy, RSV, and influenza virus were 145, 121, and 81, and the corresponding DALYs were 13,307, 11,207, and 7,358 (Table 6; Figure 2A). The hospital cohort separately showed that RSV, human rhinovirus, influenza virus, and M. pneumoniae were commonly detected in clinical testing of recent pediatric pneumonia hospitalizations (Figure 2B).

**Figure 2:**
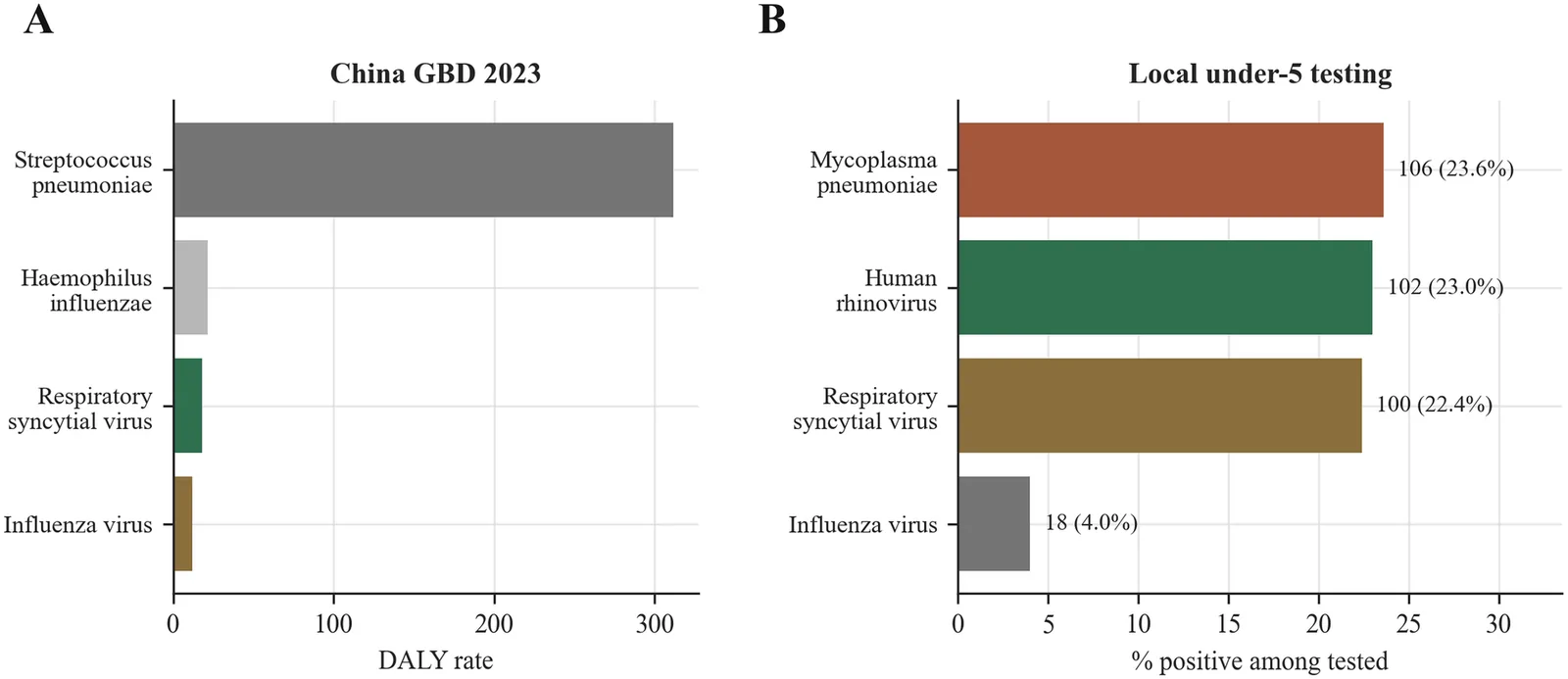
Population-level pathogen burden and hospital testing results in China. **(A)** GBD 2023 pathogen-attributed DALY rates for China. **(B)** Laboratory positivity among tested hospitalized children younger than 5 years. The panels use different data sources and denominators and are not a validation comparison.

### Pathogen-related burden and SDI differences

Among the pathogens included in this analysis, S. pneumoniae was the vaccine-relevant bacterial pathogen causing the largest under-5 LRI mortality and DALY burden in 2021. In 2021, 166,379 global under-5 LRI deaths were attributed to S. pneumoniae, accounting for 33.1% of all LRI deaths; the corresponding DALYs were 14,794,643, accounting for 33.0% of all LRI DALYs (Figure 3; Table 2). RSV and influenza virus also caused important viral LRI burden, but contributed less than S. pneumoniae to burden differences across SDI quintiles (Figure 4; Table 3).

**Figure 3:**
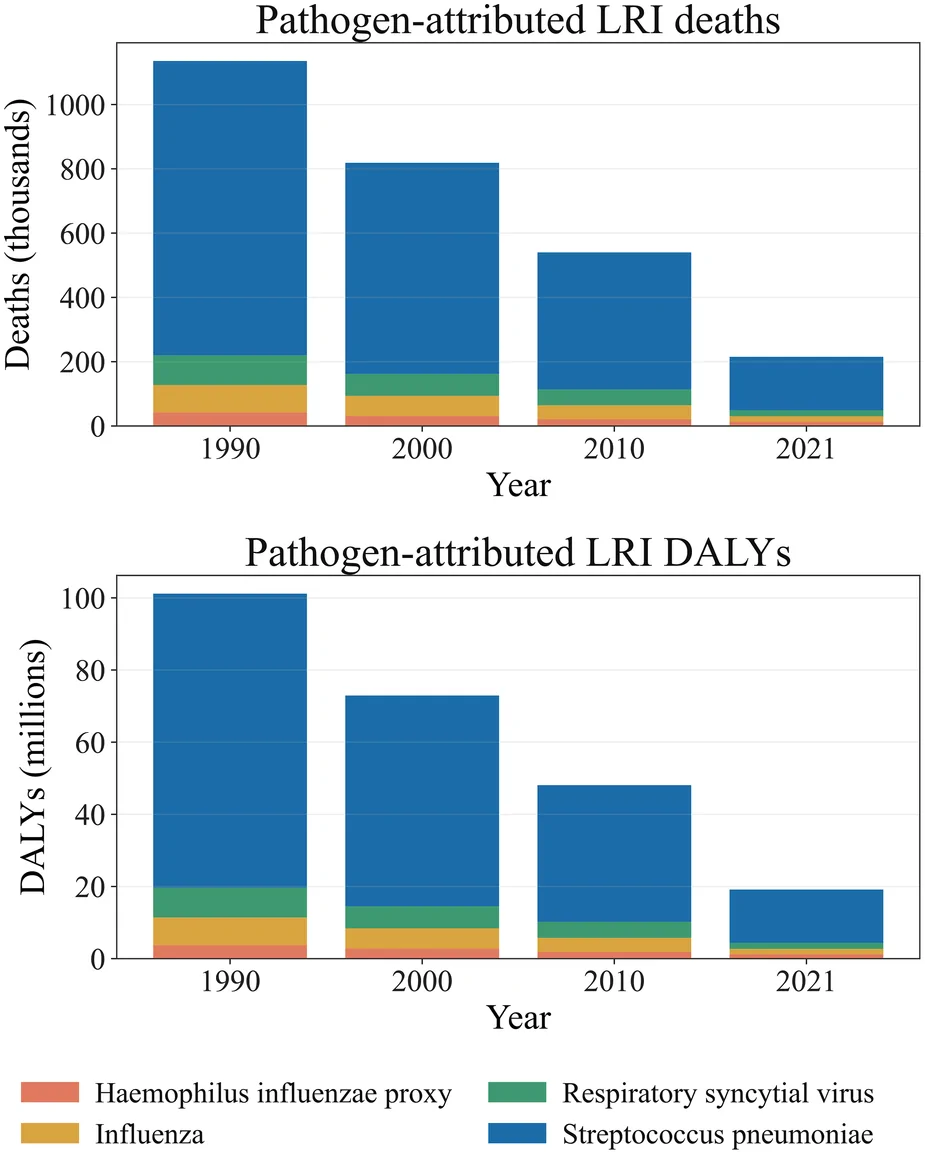
Pathogen-related burden. Changes in deaths and DALYs attributable to S. pneumoniae, H. influenzae-related burden, RSV, and influenza virus.

**Table 2:** Major pathogen burden and long-term change.

Pathogen	Deaths in 1990	Deaths in 2021	DALYs in 1990	DALYs in 2021	Share of deaths in 2021 (%)	Mortality-rate EAPC (%)
Streptococcus pneumoniae	915,372	166,379	81,472,789	14,794,643	33.1	−5.65
RSV	92,228	18,369	8,262,532	1,642,093	3.6	−3.55
Influenza virus	85,962	16,811	7,684,151	1,498,785	3.3	−3.83
Haemophilus influenzae proxy	41,747	13,506	3,739,323	1,209,901	2.7	−4.13

RSV, respiratory syncytial virus; DALY, disability-adjusted life-year; EAPC, estimated annual percentage change.

**Figure 4:**
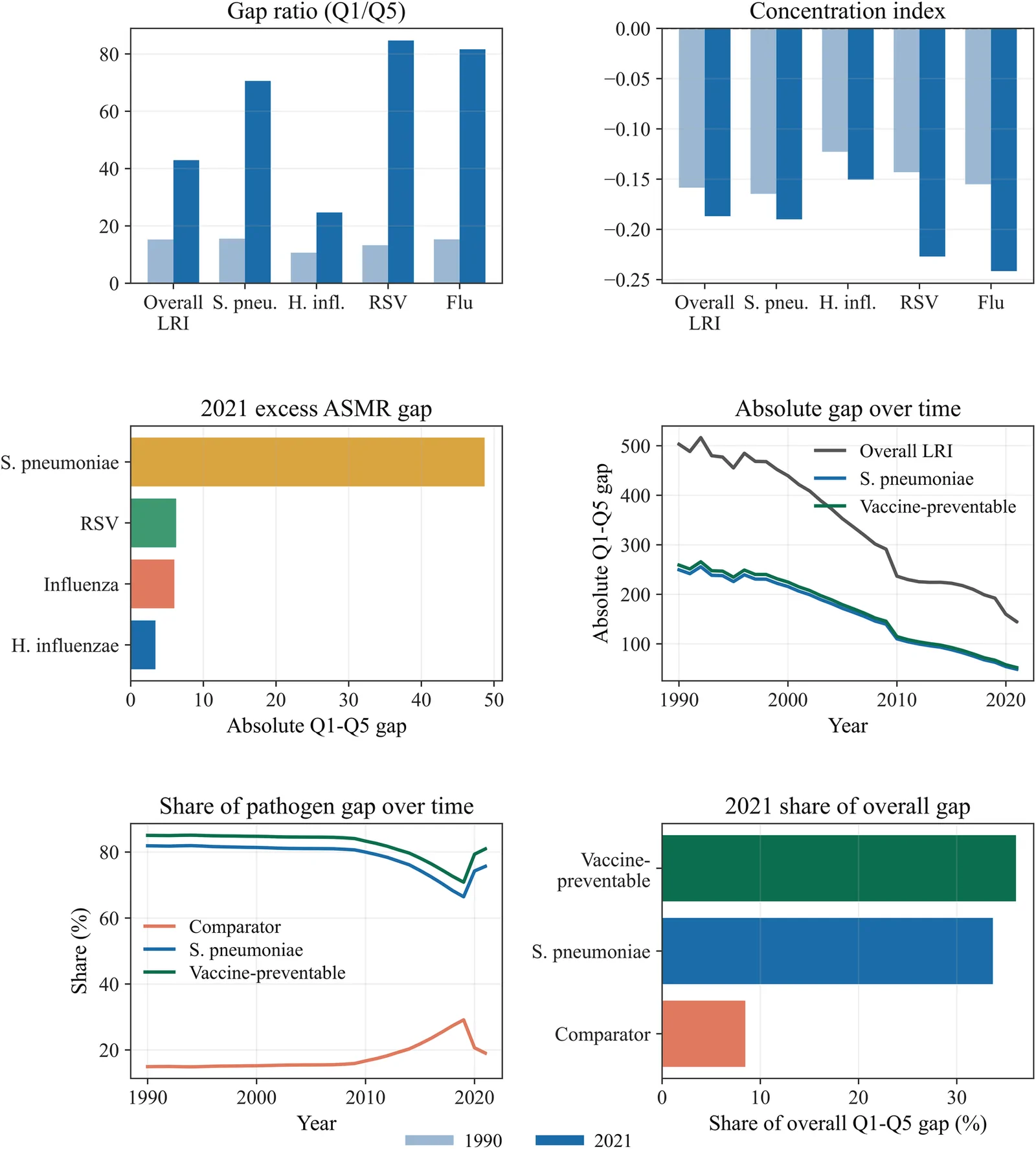
Contribution of pathogen-related burden to under-5 LRI inequality. Pathogen-related mortality-rate differences, concentration indices, and inequality decomposition across SDI quintiles.

**Table 3:** Inequality in age-standardized mortality rates for under-5 LRI and major pathogens between SDI quintiles.

Year	Outcome	Mean ASMR in lowest SDI quintile	Mean ASMR in highest SDI quintile	Q1:Q5 ratio	Slope index of inequality	Concentration index
1990	Overall LRI	538.4	35.3	15.26	−411.1	−0.158
1990	Streptococcus pneumoniae	266.7	17.1	15.56	−206.3	−0.165
1990	Haemophilus influenzae proxy	10.6	1.0	10.67	−6.7	−0.123
1990	RSV	24.9	1.9	13.26	−17.8	−0.143
1990	Influenza virus	24.1	1.6	15.30	−18.2	−0.155
2021	Overall LRI	148.1	3.4	42.92	−144.6	−0.187
2021	Streptococcus pneumoniae	49.4	0.7	70.56	−49.7	−0.190
2021	Haemophilus influenzae proxy	3.5	0.1	24.69	−3.0	−0.150
2021	RSV	6.3	0.1	84.67	−6.9	−0.227
2021	Influenza virus	6.1	0.1	81.61	−6.9	−0.242

Q1 denotes the lowest SDI quintile and Q5 the highest. ASMR is per 100,000 population. Negative inequality metrics indicate concentration of burden in lower-SDI countries.

When stratified by SDI quintile, countries in the lowest SDI group had substantially higher under-5 LRI mortality rates than countries in the highest SDI group (Figure 4). In 2021, among the pathogens included in this study, S. pneumoniae was the main contributor to this gap: it accounted for 75.7% of the summed pathogen-specific excess age-standardized mortality-rate difference between the lowest and highest SDI quintiles, and S. pneumoniae together with H. influenzae-related burden accounted for 81.0%. When referenced to the overall LRI age-standardized mortality-rate gap, S. pneumoniae and H. influenzae-related burden together accounted for 36.0%, whereas RSV and influenza virus together accounted for 8.5% (35). This indicates that SDI-related differences in LRI burden are not distributed evenly across pathogen classes, but are concentrated more strongly in bacterial pathogens closely tied to conjugate-vaccine policy.

Inequality analyses further showed that, although under-5 LRI mortality rates had declined substantially since 1990, relative gaps across SDI groups had not narrowed. In 1990, the mean overall LRI age-standardized mortality rate was 538.4 per 100,000 in the lowest SDI quintile and 35.3 per 100,000 in the highest SDI quintile, giving a low-to-high SDI ratio of 15.26. By 2021, the corresponding rates had fallen to 148.1 and 3.4 per 100,000, but the ratio had increased to 42.92. Pneumococcal mortality showed a similar gradient: in 2021, the rate was 49.4 per 100,000 in the lowest SDI quintile and 0.7 per 100,000 in the highest SDI quintile, giving a low-to-high SDI ratio of 70.56. These findings suggest that a decline in the global average burden does not necessarily mean that gaps between countries have converged; the remaining mortality risk remains strongly concentrated in countries with lower social development.

### Immunization coverage, vaccine-introduction timing, and ecological associations

WHO/UNICEF immunization data further showed that SDI groups with higher vaccine-relevant bacterial burden also tended to have lower vaccine coverage and later vaccine introduction. In 2021, Hib3 coverage was 82.6% in the lowest SDI quintile and 94.4% in the highest SDI quintile; PCV3 coverage was 79.5% and 91.2%, respectively (Figure 5B). Vaccine-introduction timing also differed: the median year of first Hib3 introduction was 14 years later in the lowest than in the highest SDI quintile, and PCV3 introduction was 6 years later (Figure 5A). This suggests that countries carrying higher vaccine-relevant bacterial LRI burden are also often countries with relatively delayed vaccine introduction and inadequate sustained coverage.

**Figure 5:**
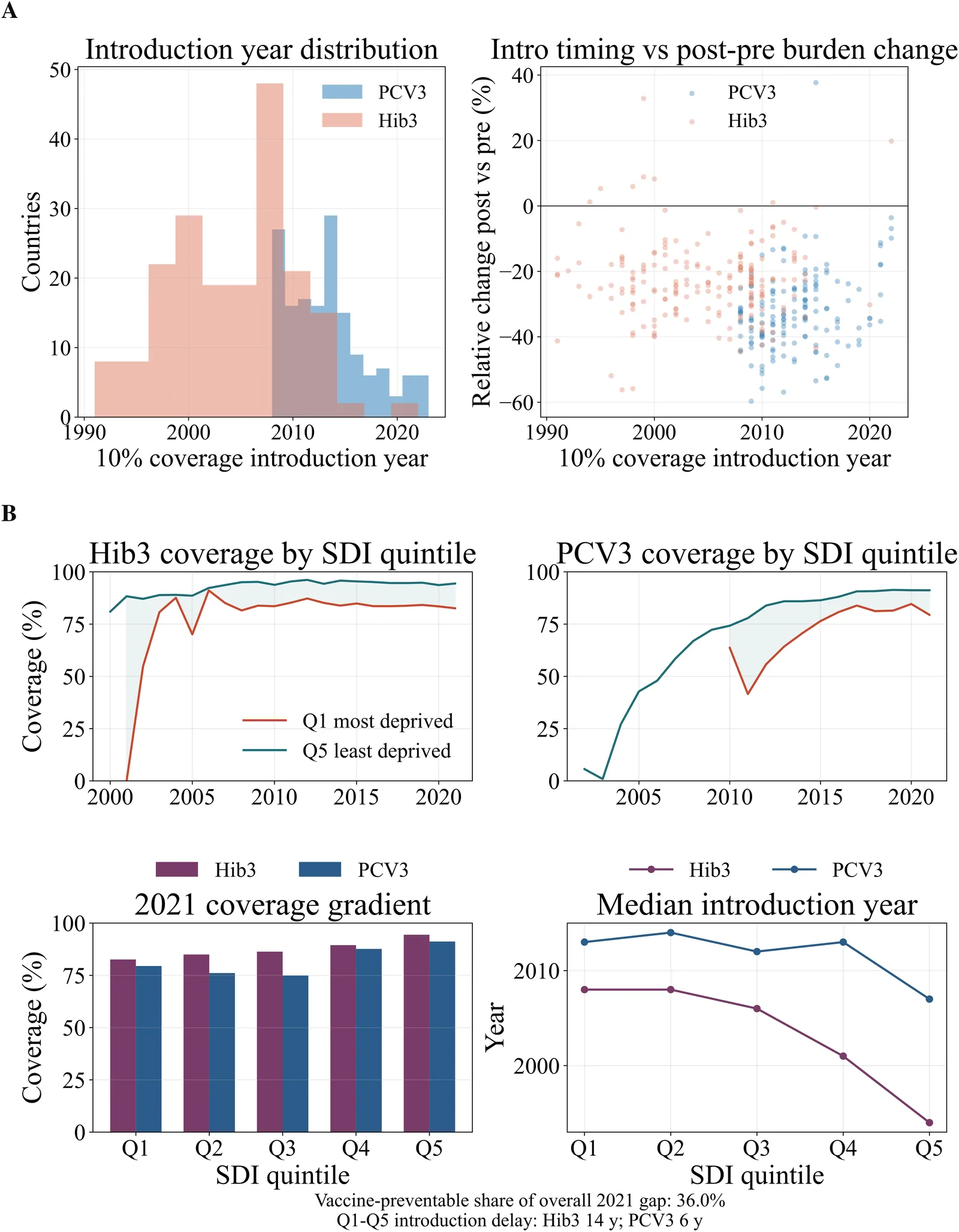
Hib3 and PCV3 programme inequalities across SDI groups. **(A)** Hib3 and PCV3 introduction timing and its relationship with SDI. **(B)** Coverage levels and coverage gaps across SDI groups.

Fixed-effects models included only country-years with complete information on vaccine coverage, pathogen burden, and covariates, and therefore used smaller samples than the descriptive analysis panel. The PCV3 models included 465 country-year observations from 62 countries between 2000 and 2021, and the Hib3 models included 1,111 country-year observations from 81 countries. In the prespecified 1-year lag models, each 10-percentage-point increase in PCV3 coverage was associated with a 0.9% relative decrease in pneumococcal age-standardized mortality and DALY rates (95% CI −1.9 to 0.2), although the confidence interval crossed zero. Each 10-percentage-point increase in Hib3 coverage was associated with a 1.8% relative decrease in age-standardized mortality and DALY rates for the H. influenzae proxy (95% CI −3.4 to −0.2) (Figure 6A; Table 4). However, across the prespecified 16 tests, the main PCV3 and Hib3 results did not remain significant after FDR correction at 0.05, and no result passed Bonferroni correction. The fixed-effects models were therefore broadly consistent in direction with the descriptive findings, but support only country-level association, not causal vaccine-effect estimation. The lag profile is shown in Figure 6B; rollout and diagnostic sensitivity results are in Supplementary Figure S2.

**Figure 6:**
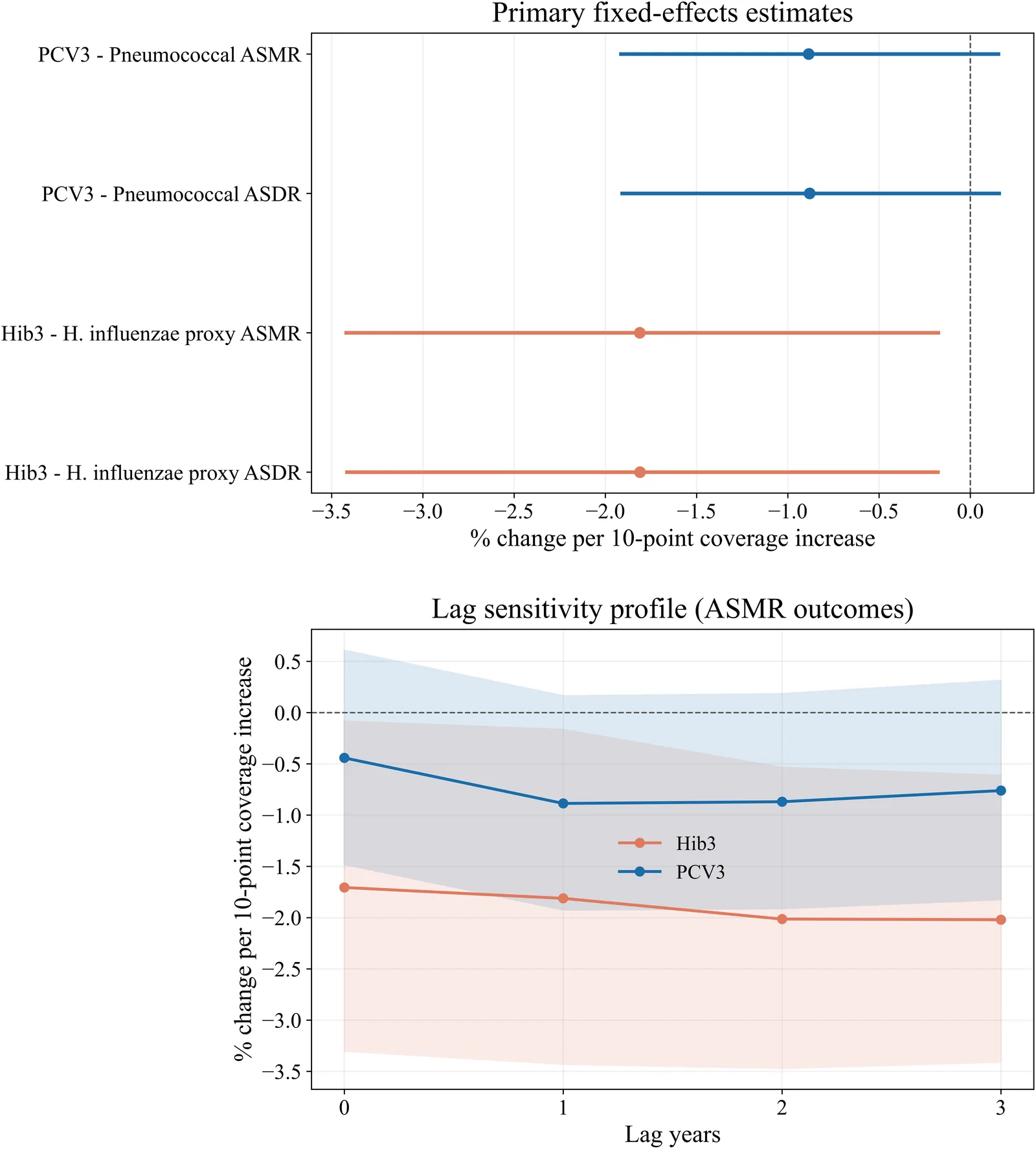
Primary fixed-effects estimates and lag sensitivity profiles for vaccine coverage and pathogen-related burden. **(A)** Primary fixed-effects estimates. **(B)** Lag sensitivity profiles for ASMR outcomes. Results are ecological associations, not vaccine-effect estimates.

**Table 4:** Main fixed-effects models of Hib3 and PCV3 coverage and pathogen-related burden.

Vaccine indicator	Outcome	Change per 10-percentage-point coverage increase	95% CI	*P* value	FDR-adjusted *P* value	Bonferroni-adjusted *P* value	Countries	Country-years
Hib3	H. influenzae proxy ASMR	−1.8%	−3.4 to −0.2	0.031	0.079	0.498	81	1,111
Hib3	H. influenzae proxy ASDR	−1.8%	−3.4 to −0.2	0.031	0.079	0.495	81	1,111
PCV3	Pneumococcal ASMR	−0.9%	−1.9 to 0.2	0.098	0.143	1.000	62	465
PCV3	Pneumococcal ASDR	−0.9%	−1.9 to 0.2	0.100	0.143	1.000	62	465

Models included country and year fixed effects, with standard errors clustered by country. Results are country-year ecological associations and are not interpreted as causal vaccine-effect estimates.

### Age stratification and sensitivity analyses

Age-stratified analyses showed that infants aged 1 to 5 months accounted for a relatively large share of under-5 LRI deaths in 2021. By contrast, associations between coverage and burden were more apparent in older children, especially those aged 2 to 4 years. This pattern suggests that LRI risk, direct vaccine protection, herd protection, and exposure opportunities differ across age groups. Young infants may carry higher mortality risk, but national-year annual coverage indicators have limited ability to show whether they received vaccination on time and may inadequately capture herd-immunity protection. Event-study, segmented interrupted time-series, controlled interrupted time-series, and synthetic-control sensitivity analyses are provided in Supplementary Figures S3, S4. Because these analyses did not fully remove concerns about pre-introduction trends, they were not used for causal inference. Counterfactual and diagnostic sensitivity estimates at year +3 are shown in Supplementary Figure S2B; these exploratory results were not used for causal inference.

## Clinical characteristics of pediatric pneumonia hospitalization data from China

To characterize clinical-care needs independently of the population estimates, we analyzed recent pediatric pneumonia hospitalization records from China. Between July 1, 2024 and December 31, 2025, 1,717 pediatric pneumonia hospitalization records were included, of which 889 (51.8%) were among children aged 0 to 4 years (Figure 7A). In the 0- to 4-year subgroup, 170 records (19.1%) were classified as severe, 184 (20.7%) involved at least one form of respiratory support, and 92 (10.3%) involved high-flow oxygen therapy, non-invasive ventilation, or invasive mechanical ventilation. The median length of stay in this subgroup was 6.0 days (Figure 7B; Table 5).

**Figure 7:**
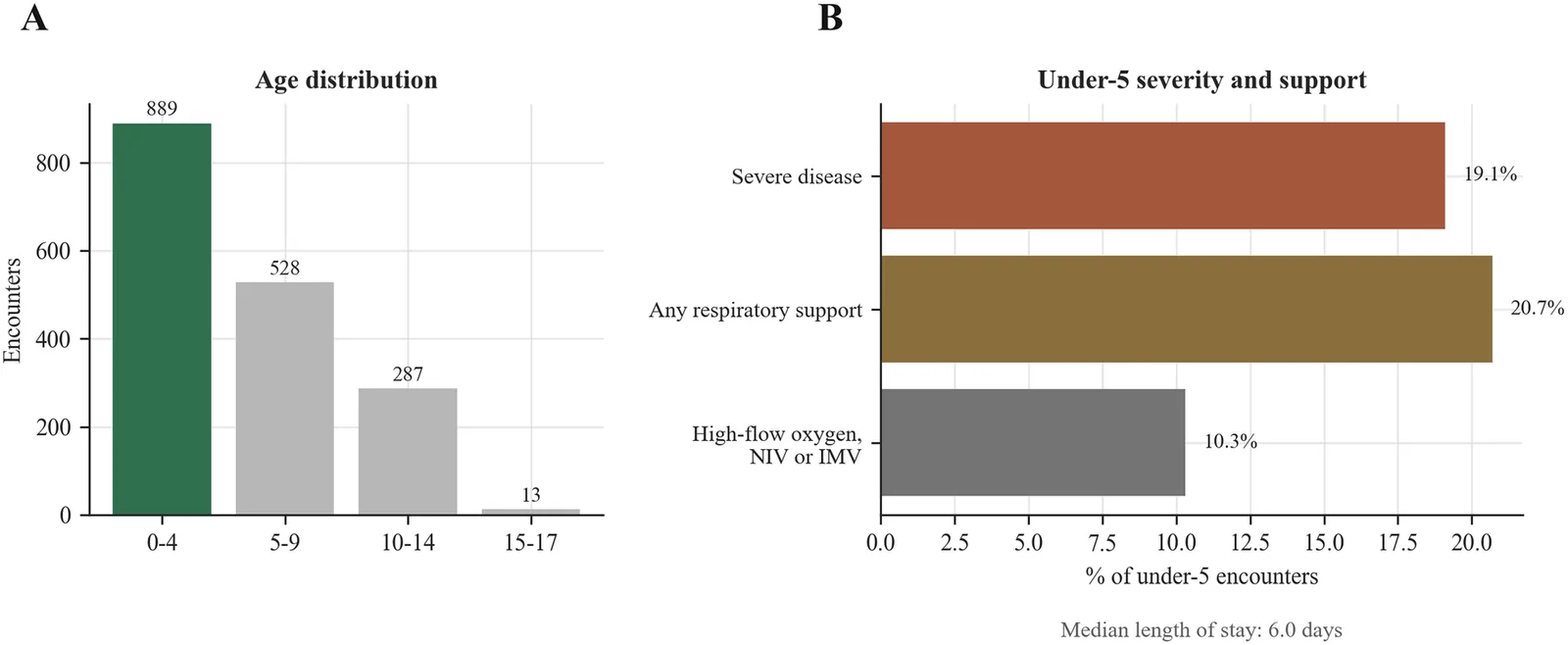
Clinical characteristics of pediatric pneumonia hospitalization records from China. **(A)** Age distribution of pediatric pneumonia hospitalizations between July 1, 2024 and December 31, 2025. **(B)** Proportions of children aged 0 to 4 years recorded as severe, receiving any respiratory support, and receiving high-flow oxygen therapy, non-invasive ventilation, or invasive mechanical ventilation, with median length of stay annotated. These single-center data have no population denominator.

**Table 5:** Clinical characteristics of pediatric pneumonia hospitalizations among children younger than 5 years in China.

Domain	Indicator	Value
Cohort size	Total pediatric pneumonia hospitalization records	1,717
Age distribution	Children aged 0 to 4 years	889 (51.8%)
Clinical severity	Recorded as severe among children aged 0 to 4 years	170 (19.1%)
Respiratory support	Received at least one form of respiratory support among children aged 0 to 4 years	184 (20.7%)
Respiratory support	Received high-flow oxygen therapy, NIV, or IMV among children aged 0 to 4 years	92 (10.3%)
Hospitalization	Median length of stay among children aged 0 to 4 years	6.0 days
Pathogen testing	Laboratory-positive Mycoplasma pneumoniae among tested children aged 0 to 4 years	106 (23.6%)
Pathogen testing	Laboratory-positive RSV among tested children aged 0 to 4 years	100 (22.4%)
Pathogen testing	Laboratory-positive influenza virus among tested children aged 0 to 4 years	18 (4.0%)
Pathogen testing	Laboratory-positive human rhinovirus among tested children aged 0 to 4 years	102 (23.0%)

NIV, non-invasive ventilation; IMV, invasive mechanical ventilation; RSV, respiratory syncytial virus. Positivity is calculated among children receiving the relevant test.

**Table 6:** China GBD 2023 pathogen estimates and hospital testing records among children younger than 5 years.

Pathogen	China GBD 2023 deaths	China GBD 2023 DALYs	Hospital under-5 tested	Hospital under-5 positives	Positivity among tested
Streptococcus pneumoniae	2,162	193,740	4	1	1 (25.0%)
Haemophilus influenzae	145	13,307	4	0	0 (0.0%)
RSV	121	11,207	446	100	100 (22.4%)
Influenza virus	81	7,358	446	18	18 (4.0%)

Hospital testing results are descriptive and were not used to evaluate GBD pathogen attribution. The GBD and hospital components use different denominators, sampling frames, and purposes of inference.

In this independent clinical cohort, children aged 0 to 4 years accounted for the largest proportion of pediatric pneumonia hospitalizations (Figure 7A), and about one fifth were classified as severe or received respiratory support (Figure 7B). Unlike GBD, hospitalization data describe bedside information such as disease severity, respiratory support, and length of stay. These variables are directly relevant to pediatric pneumonia care capacity, oxygen-system readiness, and ward resource allocation, but are not population incidence or mortality estimates.

## Pathogen test results in hospitalization records

Among tested hospitalization records for children younger than 5 years, laboratory positivity was 106/449 (23.6%) for M. pneumoniae, 100/446 (22.4%) for RSV, 102/444 (23.0%) for human rhinovirus, and 18/446 (4.0%) for influenza virus (Figure 2B; Table 6). RSV and influenza virus appear in both GBD pathogen-attribution results and hospital testing records, but the measures retain different denominators and designs. S. pneumoniae and H. influenzae were infrequently tested or recorded in the hospital cohort (Table 6); hospital testing was not used to evaluate GBD pathogen attribution.

Exploratory adjusted models showed clinically interpretable differences in severity records among hospitalized cases. Higher log-transformed C-reactive protein was associated with a greater likelihood of being recorded as severe (OR 1.22, 95% CI 1.07 to 1.39). Text-derived *M. pneumoniae* records were also associated with a greater likelihood of being recorded as severe (OR 2.07, 95% CI 1.23 to 3.49) (Table 7).

**Table 7:** Exploratory clinical model related to severe labels among pediatric pneumonia hospitalization records for children aged 0 to 4 years.

Outcome	Analysis population	Variable	OR	95% CI
Severe label	Hospitalization records aged 0 to 4 years	Log-transformed C-reactive protein	1.22	1.07 to 1.39
Severe label	Hospitalization records aged 0 to 4 years	Text-derived Mycoplasma pneumoniae record	2.07	1.23 to 3.49

This exploratory in-hospital analysis identifies severity-related signals among hospitalized cases; it was not used to build a national prediction model or infer population risk.

We further summarized linkage between hospital pneumonia hospitalization records and microbiology test results. Among under-5 hospitalization records, both the earlier and recent data had high proportions linkable to microbiology test results: 988/1,117 (88.5%) and 1,003/1,193 (84.1%), respectively. Among records linked to microbiology results, the proportions with at least one positive pathogen test were 422/988 (42.7%) and 387/1,003 (38.6%). RSV positivity was similar in the two datasets (10.2% and 10.1%); human rhinovirus positivity was 10.8% in the recent data and 9.4% in the earlier data; *M. pneumoniae* positivity was 10.8% and 7.4%, respectively.

## Discussion

This study examined a single childhood LRI problem through two parallel evidence components: population burden and clinical-care needs. The global component showed that under-5 LRI deaths and DALYs have declined substantially since 1990, but remaining burden is concentrated in low-SDI settings and weighted toward vaccine-relevant bacterial pathogens. The immunization component showed that countries with higher vaccine-relevant bacterial burden also tended to introduce Hib3 and PCV3 later and to have lower coverage; fixed-effects results were ecological associations, not estimates of vaccine effectiveness. The independent hospitalization cohort from China showed that young children accounted for a large share of admissions and that severe disease and respiratory-support needs were not uncommon. These components are not one statistical sample and do not validate each other. Together, they identify complementary prevention and care priorities for childhood LRI.

The pathogen-attribution results strengthen the central population-burden finding of this manuscript: residual LRI inequality is not spread evenly across all pathogens. *S. pneumoniae* remained the vaccine-relevant bacterial pathogen responsible for the largest under-5 LRI mortality burden in 2021, and explained most of the pathogen-specific mortality-rate gap between the lowest and highest SDI quintiles. The *H. influenzae* proxy contributed less, but together with *S. pneumoniae*, vaccine-relevant bacterial pathogens accounted for 81.0% of the pathogen-specific gap. This result is consistent with previous studies of pneumococcal and Hib burden, and with the understanding that severe childhood pneumonia in the vaccine era remains concentrated in areas with weaker prevention and treatment resources (6, 8–13).

Immunization coverage and vaccine-introduction timing further support the interpretation that burden inequality and program inequality coexist. Countries in the lowest SDI quintile had lower Hib3 and PCV3 coverage than countries in the highest SDI quintile, and introduced these vaccines later. The fixed-effects models were directionally consistent with the descriptive results, but their limitations are also clear: country and year fixed effects can control for stable country differences and common year effects, but cannot fully remove time-varying confounding from nutrition, care-seeking behavior, oxygen availability, antibiotic supply, surveillance quality, and health-system improvement (31). Staggered introduction can also produce bias when pretrends are not parallel and effects are heterogeneous (32–34). We therefore interpret the model results linking vaccine coverage and burden as ecological associations rather than estimates of vaccine effectiveness.

This point also helps explain why the ecological coefficients in this study are weaker than vaccine effects observed in clinical trials and surveillance studies. PCV trials in The Gambia and South Africa showed that pneumococcal conjugate vaccines can reduce invasive pneumococcal disease and some pneumonia endpoints (11, 12). Post-introduction surveillance studies have also observed marked declines in vaccine-type invasive disease, with indirect protection gradually emerging after infant immunization programs are established (10). By contrast, the national-level PCV3 or Hib3 coverage indicators used in this study are more distal and coarser measures of program performance, and the GBD pathogen-attributed death and DALY outcomes are broader than vaccine-type invasive disease or radiographically confirmed pneumonia. The weaker estimates in this study therefore do not conflict with previous effectiveness evidence; they reflect the distance between ecological data, annual coverage indicators, and modelled disease burden.

Vaccination timeliness and herd protection also affect the visibility of coverage-burden associations. Direct protection depends on whether children complete primary immunization at the appropriate age. Indirect protection requires high coverage sustained over time, gradually reducing carriage and transmission. A 1-year lag in annual national coverage has limited ability to capture these processes, especially timely vaccination among the youngest infants. This also explains two seemingly different findings in the age-stratified analysis: infants aged 1 to 5 months carried a high mortality burden, whereas negative associations between coverage and pathogen-related burden were easier to observe in older children. The age group with the highest mortality risk is not necessarily the group in which a national-level coverage signal is easiest to detect.

Hib-related results need even greater caution than PCV results. Inequalities in Hib3 coverage and introduction timing are clear, and they are consistent with previous evidence that invasive Hib disease declined after Hib vaccine introduction (9, 13). However, the available pathogen outcome in this study is the GBD *H. influenzae* proxy rather than a laboratory-confirmed Hib-specific endpoint. This series may be affected by non-type b *H. influenzae*, diagnostic-practice differences, and modelling attribution strategies. Hib3-related results should therefore be understood as evidence that immunization-program inequality and bacterial-related burden are distributed in the same direction, not as a direct estimate of Hib vaccine effectiveness.

The independent hospital cohort describes the clinical-care needs that population estimates cannot measure. GBD can characterize incidence, deaths, DALYs, and pathogen attribution at the population level, but cannot show whether hospitalized children need oxygen, escalate to high-flow oxygen or ventilation, how long they stay in hospital, or how inflammatory biomarkers relate to recorded severity. In this single-center cohort, children aged 0 to 4 years accounted for more than half of pediatric pneumonia hospitalizations, and about one fifth were recorded as severe or received respiratory support. These findings describe visible clinical and resource pressure in hospital care; they are not an external validation of GBD.

The clinical cohort keeps the study connected to respiratory practice without extending its inference beyond the data. Population-burden analyses are suited to mortality, DALYs, SDI gradients, pathogen attribution, and vaccine-program inequality. Hospitalization data describe care after admission, including respiratory support, recorded severity, pathogen testing, and inflammatory markers. We do not present the single-center cohort as national surveillance, do not use hospital positivity to replace GBD pathogen attribution, and do not use either source to validate the other.

For China, the two components address complementary rather than interchangeable questions. GBD results show that under-5 LRI incidence and DALYs remain present and that S. pneumoniae remains an important pathogen-attributed burden. The hospitalization cohort shows that recent pediatric pneumonia admissions still involve severe records, oxygen therapy, and ventilatory-support needs. This means that the relevant clinical burden is not captured by mortality alone, while the hospital cohort cannot estimate population burden.

Pathogen test results also need to be interpreted under separate denominators. GBD pathogen attribution, PERCH etiologic fractions, EPIC hospital detections, and hospital positivity all provide pathogen information, but their designs and denominators differ (21, 22). The hospital data showed that *M. pneumoniae*, RSV, and human rhinovirus were commonly positive among tested hospitalization records for children younger than 5 years. This pattern is consistent with previous Chinese studies showing that pathogens in pediatric community-acquired pneumonia vary by age, region, and season, and that *M. pneumoniae*, RSV, and other respiratory viruses can play important roles in hospitalized cases (23–26). RSV and influenza virus provide a natural connection between GBD results and hospital testing because they are included in GBD pathogen attribution and are common viral categories in hospitalization testing. *M. pneumoniae* and human rhinovirus, by contrast, mainly reflect clinical testing features of recent pediatric pneumonia hospitalizations in China. *S. pneumoniae* and *H. influenzae* have important population-burden meaning in GBD, but were infrequently tested or recorded in the hospitalization records, so the hospital data are not suitable for evaluating GBD attribution estimates for these pathogens.

The findings support a public-health interpretation that gives weight to both vaccination equity and pneumonia care capacity. Vaccination can reduce risk related to some bacterial pathogens, but the pathway from infection to death is also shaped by early recognition, care-seeking behavior, hypoxemia detection, oxygen supply, antibiotic availability, and referral capacity. Studies of childhood pneumonia case management, integrated community case management, and care-seeking behavior all indicate that treatment-side capacity can substantially affect pneumonia mortality risk (36–38). Oxygen-system studies and calls to treat pulse oximetry as the fifth vital sign in pediatrics also emphasize that hypoxemia recognition and oxygen availability are important components of childhood pneumonia care capacity (39–43). Childhood LRI control should therefore not be understood only as increasing vaccine coverage; it should also incorporate inpatient severe-disease recognition, oxygen systems, and referral and treatment capacity into the same intervention pathway.

This study has several limitations. First, GBD results are modelled population-level estimates, and pathogen attribution is affected by input data quality and modelling assumptions; vaccine-coverage analyses are also based on country-year data and can only reflect population-level associations, not individual vaccine effectiveness. Second, the hospitalization data came from a single center and included only hospitalized children, and therefore cannot estimate national incidence, mortality, DALYs, or severe-pneumonia incidence. Third, hospital pathogen test results were affected by testing indications, test panels, timing of testing, and completeness of documentation.

Despite these limitations, the study offers a focused two-level interpretation of childhood LRI. Population data identify where residual burden is concentrated and how it aligns with vaccine-relevant pathogen and programme inequalities. The independent clinical cohort identifies the hospital-care needs of children with pneumonia. The value of this design is not validation between datasets; it is a clearer account of how vaccine equity and pediatric respiratory-care capacity address different parts of the same LRI pathway.

## Conclusions

Global under-5 LRI burden has decreased substantially, but residual burden remains concentrated in low-SDI settings and weighted toward vaccine-relevant bacterial pathogens. Gaps in Hib3 and PCV3 coverage and vaccine-introduction timing identify vaccine equity as a prevention priority. Independently, recent pediatric pneumonia hospitalizations in China show ongoing severe-disease and respiratory-support needs among young children. Childhood LRI control should address vaccine equity alongside early recognition, oxygen capacity, referral, and inpatient pneumonia management.

## Data Availability

Public population-level data are available from the original data providers, including GBD, WUENIC, WHO Global Health Estimates, UN-IGME, UNICEF, and World Bank resources. Aggregate supplementary results are included in the Supplementary Material. Access to de-identified hospital clinical records may be requested from the corresponding author, subject to institutional review and approval.

## References

[1] Sirota SB Bender RG Dominguez R-MV Movo A Swetschinski LR Araki DT. Global burden of lower respiratory infections and aetiologies, 1990–2023: a systematic analysis for the global burden of disease study 2023. Lancet Infect Dis. (2026) 26(4):343–61. 10.1016/S1473-3099(25)00689-9 41412141

[2] McAllister DA Liu L Shi T Chu Y Reed C Burrows J. Global, regional, and national estimates of pneumonia morbidity and mortality in children younger than 5 years between 2000 and 2015: a systematic analysis. Lancet Glob Health. (2019) 7:e47–57. 10.1016/S2214-109X(18)30408-X 30497986 PMC6293057

[3] Perin J Mulick A Yeung D Villavicencio F Lopez G Strong KL. Global, regional, and national causes of under-5 mortality in 2000–19: an updated systematic analysis with implications for the sustainable development goals. Lancet Child Adolesc Health. (2022) 6:106–15. 10.1016/S2352-4642(21)00311-4 34800370 PMC8786667

[4] Bhutta ZA Das JK Walker N Rizvi A Campbell H Rudan I. Interventions to address deaths from childhood pneumonia and diarrhoea equitably: what works and at what cost? Lancet. (2013) 381:1417–29. 10.1016/S0140-6736(13)60648-0 23582723

[5] Izadnegahdar R Cohen AL Klugman KP Qazi SA. Childhood pneumonia in developing countries. Lancet Respir Med. (2013) 1:574–84. 10.1016/S2213-2600(13)70075-4 24461618 PMC9014772

[6] Wahl B O'Brien KL Greenbaum A Majumder A Liu L Chu Y. Burden of streptococcus pneumoniae and haemophilus influenzae type b disease in children in the era of conjugate vaccines: global, regional, and national estimates for 2000–15. Lancet Glob Health. (2018) 6:e744–57. 10.1016/S2214-109X(18)30247-X 29903376 PMC6005122

[7] Shattock AJ Johnson HC Sim SY Carter A Lambach P Hutubessy RCW. Contribution of vaccination to improved survival and health: modelling 50 years of the expanded programme on immunization. Lancet. (2024) 403:2307–16. 10.1016/S0140-6736(24)00850-X 38705159 PMC11140691

[8] O'Brien KL Wolfson LJ Watt JP Henkle E Deloria-Knoll M McCall N. Burden of disease caused by streptococcus pneumoniae in children younger than 5 years: global estimates. Lancet. (2009) 374:893–902. 10.1016/S0140-6736(09)61204-6 19748398

[9] Watt JP Wolfson LJ O'Brien KL Henkle E Deloria-Knoll M McCall N. Burden of disease caused by haemophilus influenzae type b in children younger than 5 years: global estimates. Lancet. (2009) 374:903–11. 10.1016/S0140-6736(09)61203-4 19748399

[10] Whitney CG Farley MM Hadler J Harrison LH Bennett NM Lynfield R. Decline in invasive pneumococcal disease after the introduction of protein-polysaccharide conjugate vaccine. N Engl J Med. (2003) 348:1737–46. 10.1056/NEJMoa022823 12724479

[11] Cutts FT Zaman SMA Enwere G Jaffar S Levine OS Okoko JB. Efficacy of nine-valent pneumococcal conjugate vaccine against pneumonia and invasive pneumococcal disease in the Gambia: randomised, double-blind, placebo-controlled trial. Lancet. (2005) 365:1139–46. 10.1016/S0140-6736(05)71876-6 15794968

[12] Klugman KP Madhi SA Huebner RE Kohberger R Mbelle N Pierce N. A trial of a 9-valent pneumococcal conjugate vaccine in children with and those without HIV infection. N Engl J Med. (2003) 349:1341–8. 10.1056/NEJMoa035060 14523142

[13] Ladhani SN. Two decades of experience with the haemophilus influenzae serotype b conjugate vaccine in the United Kingdom. Clin Ther. (2012) 34:385–99. 10.1016/j.clinthera.2011.11.027 22244051

[14] Haeuser E Byrne S Nguyen J Raggi C McLaughlin SA Bisignano C. Global, regional, and national trends in routine childhood vaccination coverage from 1980 to 2023 with forecasts to 2030: a systematic analysis for the global burden of disease study 2023. Lancet. (2025) 406:752–85. 10.1016/S0140-6736(25)01037-2 PMC12338332 40578370

[15] Evans B Kaiser L Keiser O Jombart T. Routine immunisation coverage shows signs of recovery at global level postpandemic, but important declines persist in about 20% of countries. Vaccines (Basel). (2025) 13:388. 10.3390/vaccines13040388 40333223 PMC12031510

[16] Restrepo-Méndez MC Barros AJD Wong KLM Johnson HL Pariyo G França GVA. Inequalities in full immunization coverage: trends in low- and middle-income countries. Bull World Health Organ. (2016) 94:794–805B. 10.2471/BLT.15.162172 PMC5096343 27821882

[17] Barros AJD Ronsmans C Axelson H Loaiza E Bertoldi AD França GVA. Equity in maternal, newborn, and child health interventions in countdown to 2015: a retrospective review of survey data from 54 countries. Lancet. (2012) 379:1225–33. 10.1016/S0140-6736(12)60113-5 22464386

[18] Troeger C Blacker B Khalil IA Rao PC Cao J Zimsen SRM. Estimates of the global, regional, and national morbidity, mortality, and aetiologies of lower respiratory infections in 195 countries, 1990–2016: a systematic analysis for the global burden of disease study 2016. Lancet Infect Dis. (2018) 18:1191–210. 10.1016/S1473-3099(18)30310-4 30243584 PMC6202443

[19] Burton A Monasch R Lautenbach B Gacic-Dobo M Neill M Karimov R. WHO and UNICEF estimates of national infant immunization coverage: methods and processes. Bull World Health Organ. (2009) 87:535–41. 10.2471/BLT.08.053819 19649368 PMC2704038

[20] World Health Organization, UNICEF. WHO/UNICEF Immunization Coverage Estimates—2024 Revision. Geneva: World Health Organization; 2025. Available online at: https://www.who.int/publications/m/item/WUENIC_notes (Accessed May 31, 2026).

[21] O'Brien KL Baggett HC Brooks WA Feikin DR Hammitt LL Higdon MM. Causes of severe pneumonia requiring hospital admission in children without HIV infection from Africa and Asia: the PERCH multi-country case-control study. Lancet. (2019) 394:757–79. 10.1016/S0140-6736(19)30721-4 PMC6727070 31257127

[22] Jain S Williams DJ Arnold SR Ampofo K Bramley AM Reed C. Community-acquired pneumonia requiring hospitalization among U.S. Children. N Engl J Med. (2015) 372:835–45. 10.1056/NEJMoa1405870 25714161 PMC4697461

[23] Ning G Wang X Wu D Yin Z Li Y Wang H. The etiology of community-acquired pneumonia among children under 5 years of age in mainland China, 2001–2015: a systematic review. Hum Vaccin Immunother. (2017) 13:2742–50. 10.1080/21645515.2017.1371381 28922613 PMC5703373

[24] Oumei H Xuefeng W Jianping L Kunling S Rong M Zhenze C. The etiology of community-acquired pneumonia in 1500 hospitalized children. J Med Virol. (2018) 90:421–8. 10.1002/jmv.24963 28975629 PMC7166354

[25] Qin T Zhou H Ren H Meng J Du Y Mahemut M. Incidence, etiology, and environmental risk factors of community-acquired pneumonia requiring hospitalization in China: a 3-year, prospective, age-stratified, multicenter case-control study. Open Forum Infect Dis. (2021) 8:ofab499. 10.1093/ofid/ofab499 35548172 PMC8522381

[26] You J Zhang L Chen W Wu Q Zhang D Luo Z. Epidemiological characteristics of mycoplasma pneumoniae in hospitalized children before, during, and after COVID-19 pandemic restrictions in Chongqing, China. Front Cell Infect Microbiol. (2024) 14:1424554. 10.3389/fcimb.2024.1424554 39220288 PMC11362049

[27] Stevens GA Alkema L Black RE Boerma JT Collins GS Ezzati M. Guidelines for accurate and transparent health estimates reporting: the GATHER statement. Lancet. (2016) 388:e19–23. 10.1016/S0140-6736(16)30388-9 27371184

[28] Von Elm E Altman DG Egger M Pocock SJ Gotzsche PC Vandenbroucke JP. The strengthening the reporting of observational studies in epidemiology (STROBE) statement: guidelines for reporting observational studies. Lancet. (2007) 370:1453–7. 10.1016/S0140-6736(07)61602-X 18064739

[29] World Health Organization. Global Health Estimates 2021: Disease Burden by Cause, age, sex, by Country and by Region, 2000-2021. Geneva: World Health Organization (2024). Available online at: https://www.who.int/data/global-health-estimates (Accessed May 31, 2026).

[30] United Nations Inter-agency Group for Child Mortality Estimation. Levels and Trends in Child Mortality 2024. New York: UNICEF (2024). Available online at: https://childmortality.org/ (Accessed May 31, 2026).

[31] Gunasekara FI Richardson K Carter K Blakely T. Fixed effects analysis of repeated measures data. Int J Epidemiol. (2014) 43:264–9. 10.1093/ije/dyt221 24366487

[32] Goodman-Bacon A. Difference-in-differences with variation in treatment timing. J Econom. (2021) 225:254–77. 10.1016/j.jeconom.2021.03.014

[33] Sun L Abraham S. Estimating dynamic treatment effects in event studies with heterogeneous treatment effects. J Econom. (2021) 225:175–99. 10.1016/j.jeconom.2020.09.006

[34] Callaway B Sant'Anna PHC. Difference-in-differences with multiple time periods. J Econom. (2021) 225:200–30. 10.1016/j.jeconom.2020.12.001

[35] Li Y Wang X Blau DM Caballero MT Feikin DR Gill CJ. Global, regional, and national disease burden estimates of acute lower respiratory infections due to respiratory syncytial virus in children younger than 5 years in 2019: a systematic analysis. Lancet. (2022) 399:2047–64. 10.1016/S0140-6736(22)00478-0 35598608 PMC7613574

[36] Theodoratou E Al-Jilaihawi S Woodward F Ferguson J Jhass A Balliet M. The effect of case management on childhood pneumonia mortality in developing countries. Int J Epidemiol. (2010) 39(Suppl 1):i155–71. 10.1093/ije/dyq032 20348118 PMC2845871

[37] Mosites EM Matheson AI Kern E Manhart LE Morris SS Hawes SE. Care-seeking and appropriate treatment for childhood acute respiratory illness: an analysis of demographic and health survey and multiple indicators cluster survey datasets for high-mortality countries. BMC Public Health. (2014) 14:446. 10.1186/1471-2458-14-446 24884919 PMC4024183

[38] Oliphant NP Manda S Daniels K Odendaal WA Besada D Kinney M. Integrated community case management of childhood illness in low- and middle-income countries. Cochrane Database Syst Rev. (2021) 2021:CD012882. 10.1002/14651858.CD012882.pub2 PMC8094443 33565123

[39] Duke T Wandi F Jonathan M Matai S Kaupa M Saavu M. Improved oxygen systems for childhood pneumonia: a multihospital effectiveness study in Papua New Guinea. Lancet. (2008) 372:1328–33. 10.1016/S0140-6736(08)61164-2 18708248

[40] Zar HJ McCollum ED. Pulse oximetry to detect paediatric hypoxaemia-the fifth vital sign. Lancet Glob Health. (2023) 11:e1653–55. 10.1016/S2214-109X(23)00380-7 37793415

[41] World Health Organization, UNICEF. Ending Preventable Child Deaths from Pneumonia and Diarrhoea by 2025: The Integrated Global Action Plan for Pneumonia and Diarrhoea (GAPPD). Geneva: World Health Organization (2013). Available online at: https://iris.who.int/handle/10665/79200 (Accessed May 31, 2026).

[42] World Health Organization. Oxygen Therapy for Children: A Manual for Health Workers. Geneva: World Health Organization (2016). Available online at: https://iris.who.int/handle/10665/204584 (Accessed May 31, 2026).

[43] World Health Organization. Pocket Book of Hospital Care for Children: Guidelines for the Management of Common Childhood Illnesses. 2nd edn. Geneva: World Health Organization (2013). Available online at: https://iris.who.int/handle/10665/81170 (Accessed May 31, 2026). 24006557

