# Supplementary material for: Global and China lower respiratory infection burden in children under 5 years: implications for vaccine equity and pediatric clinical care
Source: Front Med (Lausanne). 2026 Oct 22;13:1971991. doi: 10.3389/fmed.2026.1971991 (PMC13736825; doi:10.3389/fmed.2026.1971991)
Supplement: Supplementary file 1 [file Datasheet1.pdf]

Supplementary Figure Legends and Table S5 Caption

Manuscript 1971991 | DOI 10.3389/fmed.2026.1971991

Supplementary Figure S1.

Extended-year burden trends and comparison of public mortality sources. (A) Changes in deaths, DALYs, and incidence during 2022 to 2023. (B) Comparison between GBD and WHO Global Health Estimates mortality estimates, and mortality-rate differences between the lowest and highest SDI quintiles.

Supplementary Figure S2.

Vaccine rollout context and diagnostic sensitivity summary. (A) Countries with observed vaccine introduction and median 10% coverage introduction year. (B) Counterfactual and diagnostic sensitivity estimates at year +3.

Supplementary Figure S3.

Event-study and segmented interrupted time-series sensitivity analyses of pathogen-related burden changes after vaccine introduction. (A) Event-study results. (B) Segmented interrupted time-series results.

Supplementary Figure S4.

Controlled interrupted time-series and synthetic-control sensitivity analyses of pathogen-related burden changes after vaccine introduction. (A) Controlled interrupted time-series results. (B) Example synthetic-control placebo analysis.

Supplementary Table S5.

Sensitivity analysis comparing year-specific SDI quintile assignment with a fixed-2021 country grouping for selected years. Values are weighted mean age-standardized mortality rates and Q1-Q5 gaps; the table is descriptive and does not change the primary dynamic grouping.
